# A Systematic Review of Renal Perfusion in Complex Abdominal Aortic Aneurysm Open Repair

**DOI:** 10.3390/jcdd11110341

**Published:** 2024-10-25

**Authors:** Diletta Loschi, Enrico Rinaldi, Annarita Santoro, Nicola Favia, Nicola Galati, Germano Melissano

**Affiliations:** Division of Vascular Surgery, IRCCS San Raffaele Scientific Institute, Vita-Salute San Raffaele University, 20132 Milan, Italy; rinaldi.enrico@hsr.it (E.R.); santoro.annarita@hsr.it (A.S.); favia.nicola@hsr.it (N.F.); galati.nicola@hsr.it (N.G.); melissano.germano@hsr.it (G.M.)

**Keywords:** juxtarenal aortic aneurysm, suprarenal aortic aneurysm, complex abdominal aortic aneurysm, renal perfusion

## Abstract

Introduction: This systematic review aims to analyze the current literature regarding 30-day mortality and postoperative acute kidney disease (AKI) in complex abdominal aortic aneurysms (cAAAs), which included juxtarenal aortic aneurysm (JAA), suprarenal aortic aneurysm (SRAA), and type IV thoracoabdominal aortic aneurysm (TAAA) open surgery (OS), to evaluate the impact of renal perfusion on AKI and to try to define which is the best way to perform it. Methods: A literature search in PubMed and Cochrane Library was performed, and articles published from January 1986 to January 2024 reporting on JAA, SRAA, and TAAA type IV open surgery management were identified. Multicenter studies, single-center series, and case series with ≥10 patients were considered eligible. Comparisons of outcomes of patients who underwent OS for complex abdominal aortic aneurysms (cAAAs) with or without perfusion of the renal arteries were analyzed when available. The titles, abstracts, and full texts were evaluated by two authors independently. The primary outcomes included AKI and 30-day mortality rates. The new-onset dialysis rate was considered a secondary outcome. Results: A total of 295 articles were evaluated, and 21 were included, totaling 5708 patients treated for cAAAs with OS. The male patients totaled 4094 (71.7%), with a mean age of 70.35 ± 8.01 and a mean renal ischemia time of 32.14 ± 12.89 min. Data were collected and analyzed, at first in the entire cohort and then divided into two groups (no perfusion of the renal arteries—group A vs. selective perfusion—group B), with 2516 patients (44.08%) who underwent cAAAs OS without perfusion of the renal arteries and 3192 patients (55.92%) with perfusion. In group B, four types of renal perfusion were reported. Among the 21 studies included, 10 reported on selective renal perfusion in cAAA OS, with several types of fluids described: (1) “enriched” Ringer’s solution, (2) “Custodiol” (Istidine-tryptophan-ketoglutarate or Custodiol HTKsolution), (3) other cold (4 °C) solutions (i.e., several combinations of 4 °C isotonic heparinized balanced salt solution containing mannitol, sodium bicarbonate, and methylprednisolone), and (4) warm blood. Thirty-day mortality for patients in group A was 4.25% (107/2516) vs. 4.29% (137/3192) in group B. The reported incidence of AKI and new onset of dialysis was, respectively, 22.14% (557/2516) and 5.45% (137/2516) for group A and 22.49% (718/3192) and 4.32% (138/3192) for group B. A total of 579 patients presented with chronic kidney disease (CKD) at admission across all studies, which included 350 (13.91%) in group A vs. 229 (7.17%) in group B. Acute kidney injury, 30-day mortality, and new-onset dialysis rate were reported in four subgroups: (1) In the “Ringer” group, 30-day mortality was 2.52% (3/113), AKI affected 27.73% (33/119) of patients, and the new-onset dialysis rate was 2.52% (3/113). (2) In the “Custodiol” group, 30-day mortality was 3.70% (3/81), AKI affected 20.17% (24/81) of patients, and the new-onset dialysis rate was 2.46% (2/81). (3) In the “cold solutions” group (i.e., NaCl and mannitol), 30-day mortality was 4.38% (130/2966), AKI affected 21.81% (647/2966) of patients, and the new-onset dialysis rate was 4.48% (133/2966). (4) In the “Warm blood” group, 30-day mortality was 3.85% (1/26), AKI affected 53.84% (14/26) of patients, and the new-onset dialysis rate was 0% (0/26). Conclusions: This systematic review highlights the lack of standard definitions for AKI, CKD, and the type of renal perfusion. Despite similar results in terms of AKI and 30-day mortality, renal perfusion seems to be protective of the new-onset hemodialysis rate. Moreover, Custodiol appears to have lower rates of AKI and hemodialysis than the other perfusion types. A prospective randomized controlled trial to perform further subgroup analysis and research the various types of renal perfusion may be necessary to identify possible benefits.

## 1. Introduction

**Figure 1:**
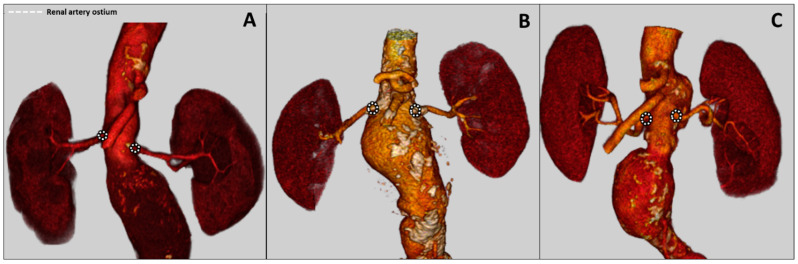
Classification of complex abdominal aortic aneurysms. (**A**) Juxtarenal aneurysm; (**B**) pararenal aneurysm; (**C**) suprarenal aneurism.

In cases of surgical management of patients with complex abdominal aortic aneurysm (cAAA) (Figure 1), suprarenal or supravisceral aortic cross-clamping may be required. Temporary renal artery hypoperfusion may lead to an increased risk of postoperative acute kidney injury (AKI), in some cases requiring (temporary or permanent) hemodialysis [1,2]. The incidence of AKI after JAA and SRAA repair is between 15% and 32.5% and is associated with increased morbidity and mortality [3]. Several risk factors for postoperative AKI have been identified, including advanced age, coronary artery disease (CAD), blood transfusion requirement, and preoperative renal dysfunction [4,5]. Currently, there are no specific recommendations provided by either the European Society for Vascular Surgery (ESVS) or the Society of Vascular Surgery (SVS) guidelines on the need for perfusion during JAA/SAA treatment and thus also on the type of perfusion [6].

The aim of this systematic review is to analyze the current literature and, by evaluating postoperative AKI and 30-day mortality after open surgery in cAAA, to understand if renal perfusion is protective of renal function, and which type of perfusion has better outcomes.

## 2. Methods

### 2.1. Search Strategy and Study Design

This systematic review was performed according to the Preferred Reporting Items for Systematic Reviews and Meta-Analyses (PRISMA) statement standards [7]. Two different Vascular Surgeons (A.S. and N.F.) performed an extensive online search on different databases (PubMed/Medline and Cochrane Library) for all the relevant studies published from January 1986 to January 2024 in the English literature which report the OS and outcomes of JAA, SRAA, and extent IV TAAA.

The following combination of MeSH terms and keywords was used: (((“juxtarenal aneurysm” [All Fields] OR “pararenal aneurysm” [All Fields]) AND (“aorta” [All Fields] AND (“juxtarenal” [All Fields] OR “pararenal” OR “infrarenal”)) OR (“juxtarenal aneurysm” [All Fields]) OR (“aneurysm” [All Fields] AND “pararenal” [All Fields]) OR (“IV type” AND “aneurysm” [All Fields]) OR (“IV type” [All Fields] AND “thoracoabdominal” [All Fields]) OR ((“renal” [All Fields] AND “perfusion” [All Fields]) OR (“renal” [All Fields] AND “protection” [All Fields]) OR (“kidney” [All Fields] AND “protection” [All Fields]) OR ((“perfusion” [All Fields] OR “protection” [All Fields]) AND (“cold” [All Fields]) OR ((“perfusion” [All Fields] OR “protection” [All Fields]) AND “Ringer’s solution” [All Fields]) OR ((“perfusion” [All Fields] OR “protection” [All Fields]) AND “Custodiol solution”))) NOT endograft NOT fenestrated NOT branched. The electronic search was supplemented and expanded using the “related articles” function of the search engine and a manual search of the relevant articles. In addition, the reference lists of all the included studies were examined for further relevant studies’ identification. The final search was run in January 2024.

### 2.2. Eligibility Criteria and Study Selection

The study selection and systematic review were performed by the authors. The titles and abstracts of the selected studies were reviewed independently by two authors (A.S. and N.F.) considering the following eligibility criteria: observational studies, multicenter studies, single-center series, and case series with more than 10 patients. No previously reported systematic review or meta-analysis was found covering this topic. Only one randomized clinical trial (RCT) including extent IV TAAAs was found on this topic [8].

The exclusion criteria included studies with relevant missing data such as patient characteristics, treatment modalities, and outcomes. Studies reporting on ruptured cAAAs were excluded because of the potential bias of the emergency setting. When publications on the same patient sample were identified or study populations overlapped, only the most recent article was included. Articles in languages other than English were excluded. The full texts of the remaining studies were obtained, and two authors (A.S. and N.F.) independently read and made a final selection of the relevant studies. The discrepancies between the authors during the search and selection were resolved by consensus with the consultation of a third author (D.L.).

The primary outcomes were defined as 30-day mortality and AKI (including the AKI classification used in the study). The acute kidney injury definition varied widely across the included studies, and for this reason all the criteria for postoperative AKI definition are specified in tables (Table 1a,b). The secondary outcome includes new-onset hemodialysis within 30 days of the operation.

### 2.3. Data Extraction

The following data were extracted and inserted into a computerized database: publication year, study design, number of patients included, age, gender, comorbidities, aneurysm size and location, perfusion (if performed and which fluid was used), postoperative AKI, new-onset hemodialysis (temporary or permanent), and 30-day mortality. The criteria for CKD and AKI are specified in tables. In the articles considered, the criteria for postoperative renal dysfunction were defined in each individual article, preferably but not exclusively based on the RIFLE classification. The RIFLE system characterizes renal dysfunction into the risk of renal dysfunction, kidney injury, insufficiency or loss of renal function, and end-stage renal disease (Table 2). The mildest stage of acute renal dysfunction is defined as an acute increase in the serum creatinine level of 1.5 mg/dL, a decrease in the glomerular filtration rate (GFR) of 25%, or a urine output of 0.5 mL/kg/h for 6 h [9] (Table 3). The incidence of temporary or permanent hemodialysis was evaluated. If the studies described combined the results for SRAA, JAA, TAAA IV type; ruptured and non-ruptured aneurysms; or with renal perfusion and without renal perfusion, only the data by single aneurysm type were extracted when possible. The data were considered unrecoverable if outcomes were reported together.

PRISMA

**Table 3:** The Aneurysm Renal Injury Score (ARISe); adapted from Twine et al. RRT: renal replacement therapy.

	Classification Creatinine/GRF	
Stage		Urine Output
1	Rise in SCr >0.3 mg/dL or <50% of baseline within 48 h	<0.5 mL/kg/h for >6 h
2	From 50% to 99% rise in SCr from the preoperative value within 7 days	
3	>2× rise in serum creatinine from the preoperative value within 7 days	
4	Need for RRT	
5	Permanent RRT	

### 2.4. Summary Process and Reporting of Results

The results, including baseline characteristics and primary and secondary endpoints, are reported and compared in Table 1a,b as described in the original articles or summarizing (mean ± standard deviation and proportions) the available data extracted by each study, according to the perfusion performed (whenever possible). The pooled mortality rate is reported as presented in the original articles.

## 3. Results

**Figure 2:**
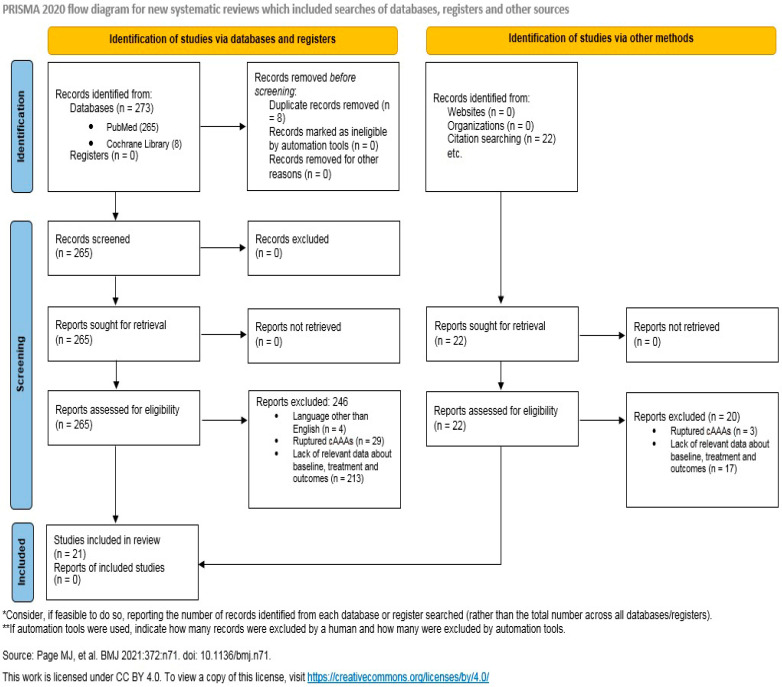
PRISMA flow diagram of studies identified and included regarding cAAAs with and without perfusion of the renal arteries.

A total of 273 papers were initially identified through the literature search, and 22 papers were added after identification through other sources (reference lists of the included studies). Eight papers were duplicated and excluded. Overall, 287 papers were screened and assessed for eligibility, and 266 were excluded because of their study design. Twenty-one studies were included in the present analysis (Figure 2). A total of 5708 patients who underwent cAAAs OS were identified. The number of male patients was 4094/5578 (73.4%); this number was calculated from the data obtained from 17/21 studies, due to the lack of data about male/female percentages in 4 studies. The mean age was 70.35 ± 8.01 and was calculated from the data obtained from 12/21 studies (4825 patients), due to the lack and heterogeneity of data about age in 9 studies. The mean renal ischemia time was 32.14 ± 12.89 min, and it was calculated from the data obtained from 10/21 studies (740 patients), due to the lack and heterogeneity of data about renal ischemia time in 11 studies. All data about the renal ischemia time, as presented in means and medians by the single study included, are shown in Table 1a,b.

The data were collected and analyzed at first in the entire cohort, and then divided into two groups:

Group A: no perfusion of the renal arteries, with 2516/5708 patients included (44.07%).

Group B: selective perfusion of the renal arteries, with 3192/5708 patients included (55.93%).

The definition of preoperative renal insufficiency varied among the studies from a serum creatinine level of 1.25 mg/dL to 2.0 mg/dL. Chronic kidney disease at admission was found in 10.14% (579/5708) of the patients across all studies included: 13.91% (350/2516) in group A vs. 7.59% (229/3014) in group B. The chronic kidney disease proportion in group B was calculated from the data obtained from 6/10 studies (3014 patients), due to the lack of data about preoperative renal insufficiency in 4 studies.

In all the included studies, the incidence of postoperative AKI was reported (data shown in Table 1). The studies defined postoperative renal dysfunction based on GFR, urine output criteria, and serum creatinine levels. Various definitions for postoperative AKI were used, as are shown in Table 1a,b.

The data about 30-day mortality were collected from all the included studies. The reported incidence of 30-day mortality for studies in group A was 4.25% (107/2516) vs. 4.29% (137/3192) in group B.

The reported incidence of postoperative AKI after elective cAAAs OS varied widely among the studies included. In group A, the reported incidence of postoperative AKI was 22.14% (557/2516), and in group B it was 22.49% (718/3192).

In most studies, renal perfusion was performed only when prolonged renal ischemia was anticipated, according to the procedure complexity [8,10,11,12,13,14,15,16,17,18,19]. In one study, renal perfusion was performed only in patients with preoperative CKD [11].

In all the included studies, postoperative new onset of temporary or permanent hemodialysis was present. The reported incidence of new onset of dialysis was 5.45% (137/2516) for the studies in group A and 4.32% (138/3192) for the studies in group B.

### 3.1. Type of Fluids Used for Selective Renal Perfusion

Among the 21 studies included, 10 reported on selective renal perfusion in cAAA OS (3192 patients), with several types of fluids described:

(1) “Enriched” Ringer’s solution (Ringer’s lactate solution enriched with 125 mg per liter of methylprednisolone and 12.5 g per liter of mannitol) [8,10,11,12,17];
(2) “Custodiol” (Istidine-tryptophan-ketoglutarate or Custodiol HTKsolution) [8,12,17];
(3) Other cold (4 °C) solutions (i.e., several combinations of 4 °C isotonic heparinized balanced salt solutions containing mannitol, sodium bicarbonate, and methylprednisolone) [13,14,15];
(4) Warm blood solutions [16].

### 3.2. Operative Technique

A complex abdominal aortic aneurysm repair can be performed using supravisceral (above the level of the superior mesenteric artery or celiac trunk), suprarenal (above the level of both renal arteries), or interrenal (above the level of one renal artery). The cross-clamping position for cAAAs was recorded in 20/21 studies. In one study, data about the clamp position were not recorded [20], and for this reason the patients were not included in this subanalysis. In group A, supraceliac cross-clamping was used in 31.82% (373/1172) of patients, suprarenal in 61.77% (724/1172), and interrenal in 6.39% (75/1172). In group B, supraceliac cross-clamping was used in 51.05% (194/380) of patients, suprarenal in 28.68% (109/380), and interrenal in 20.26% (77/380).

### 3.3. AKI Incidence, 30-Day Mortality, and Hemodialysis According to Perfusion Type Used

A subanalysis among the four subgroups including the following types of perfusion fluid used was conducted: enriched Ringer’s solution (119 patients, four studies); Istidine-tryptophan-ketoglutarate (Custodiol) (81 patients, three studies); cold solutions (i.e., NaCl and mannitol) (2966 patients, four studies); the use of warm blood perfusion, which was described in one study and selectively used in 26 patients (Table 4).

The acute kidney injury, 30-day mortality, and new-onset dialysis rates were reported in these four subgroups as follows: In the “Ringer” group, AKI affected 27.73% (33/119) of the patients, dialysis 2.52% (3/119), and the 30-day mortality rate was 3/119 (2.52%). In the “Custodiol” group, AKI affected 20.17% (24/81) of the patients, dialysis 2.46% (2/81), and the 30-day mortality rate was 3.7% (3/81). In the “cold solutions” group (i.e., NaCl and mannitol), AKI affected 21.8% (647/2966) of the patients, dialysis 4.48% (133/2966) of the patients, and the 30-day mortality rate was 4.38% (130/2966). In the “Warm blood” group, AKI affected 53.84% (14/26) of the patients, dialysis 0% (0/26), and the 30-day mortality rate was 3.85% (1/26) (Table 2).

## 4. Discussion

A recently published scoping review [21] focused on the impact of renal perfusion and different types of renal perfusion on postoperative AKI and 30-day mortality after cAAA, and, highlighting the need to standardize the definitions of AKI and CKD, showed that preoperative factors (CKD) and intraoperative technical factors (aortic cross-clamping site) have emerged as critical issues during cAAA OS that could affect outcomes and determine the possible indication for renal perfusion.

Controversy still exists on the role of the aortic cross-clamping site in the pathogenesis of postoperative AKI. Some studies report that the aortic clamping site affects serum creatinine levels in the postoperative period [22,23], whereas other studies suggest that the clamping site is not an independent risk factor for postoperative AKI or operative death [11,24,25]. This review showed that supraceliac cross-clamping was used more frequently in group B than in group A (194/380, 51.05% vs. 373/1172, 28.32%, respectively). On the other hand, the duration of aortic cross-clamping is a known risk factor for postoperative AKI. In most of the studies included in our review, renal perfusion was performed only when prolonged renal ischemia was anticipated, according to the procedure complexity [9,11,26]. In this review, the renal ischemia time is longer in group B than in group A, but, despite this finding, the incidence of postoperative AKI did not differ significantly between the two groups, being 22.14% (557/2516) in group A and 22.49% (718/3192) in group B, respectively. The reported incidence of new-onset dialysis was 5.45% (137/2516) in studies of group A and 4.32% (138/3192) in group B. These data suggest that when a complex procedure was anticipated or in cases of preoperative CKD, operators prefer the use of renal perfusion. This makes us reasonably believe that perfusion has a protective effect on kidney function.

The homogeneous data regarding the specific protocols for renal perfusion during cAAAs are lacking in the literature, and different types of perfusions were used in the included studies: warm blood, Ringer’s solution, cold solutions, and Custodiol^®^ (Dr Franz-Kohler Chemie GmbH; Bensheim, Germany) [17].

Several studies reported conflicting results on renal perfusion use: Positive results were reported by O’Donnell et al. [23], who found that in a wide range of patients, cold renal perfusion reduced the risk of AKI when the aortic clamp time exceeded 25 min (Odds Ratio 0.4 [0.2–0.97]; *p* = 0.041). In another study, Yeung et al. [15] routinely used cold saline renal perfusion in 23 consecutive patients undergoing renal revascularization, and none of them experienced postoperative elevation in serum creatinine levels. Hirose et al. [11] reported non-significant data, with no difference in the incidence of postoperative AKI and 30-day mortality between the perfused and non-perfused group. In our study, among the three types of cold perfusion, there are no clear data that favor one solution type. However, although not statistically significant, dialysis and mortality rates are low in the Ringer and Custodiol group, and these data come mainly from randomized controlled trials. An interesting finding, although mainly derived from retrospective studies, is that the incidence of dialysis in the cold solution group (mannitol/NaCL) is relatively higher than in the other groups; however, it should be noted that this group was the largest, with a total of 647 patients.

Given this uncertain scenario regarding the role of perfusion, one can turn to the literature on TAAAs, where a randomized controlled trial (the CURITIBA trial) showed better results in preventing AKI for Custodiol compared to Ringer’s solution during TAAA open surgery [8].

## 5. Limitations

One of the limitations of this study was the differing definitions of preoperative CKD across the included articles. This systematic review is limited by the relatively small number of studies providing adequate intraoperative and postoperative information and the retrospective design of all included studies; no randomized controlled trial comparing perfused and non-perfused patients during cAAA OS was available in the literature; the heterogeneity of patients’ preoperative and intraoperative factors that could impact outcomes and drive several biases; the long study period considered, whereby new surgical techniques, adjuncts, and postoperative care may have improved the outcomes; and the fact that the heterogeneity in solution and volume used for renal perfusion and the lack of a specific protocol did not allow us to draw any conclusions on which solution could better prevent postoperative AKI in cAAA OS.

## 6. Conclusions

This systematic review highlights the lack of standard definitions of AKI, CKD, and the type of cold perfusion. Considering the time of renal ischemia, renal perfusion is protective of AKI incidence, hemodialysis, and 30-day mortality. A Custodiol solution appears to have lower rates of AKI and hemodialysis than the other perfusion types.

A prospective randomized controlled trial to perform further subgroup analysis and research the various types of renal perfusion may be necessary to fully assess its benefit on postoperative renal function.

## Figures and Tables

**Table 1:** (**a**) Studies with perfusion of the renal arteries. (**b**) Studies without perfusion of the renal arteries.

**(a)**								
**Author**	**Year**	**n. pt**	**Preoperative CKD** **(%)**	**Renal Ischemia Time**	**Criteria Used for AKI Definition**	**Postoperative** **AKI** **(%)**	**Dialysis** **(%)**	**30-Day Mort** **(%)**
Chiesa	2006	34	9 (26.47)	37.3 ± 8	CREA increased > 1 mg/dL	9 (26.47)	1 (2.94)	1 (2.94)
Yeung	2008	23	12 (52.17)	37.2 ± 11.7 (25–60)	CREA increased 0.5 mg/dL	0	0	0
Pichlmaier	2008	26	8 (30.77)	45.5 (17–100)	CREA increased 0.5 mg/dL	14 (53.85)	0	1 (3.85)
Tshomba (RINGER)	2014	13	NR	39.61 ± 25.61	RIFLE	8 (61.54)	1 (7.69)	0
Tshomba (CUSTODIOL)	2014	11	NR	46.15 ± 25.61	RIFLE	6 (54.55)	0	0
Kabbani	2014	50	NR	NR	AKIN	40 (80.0)	2 (4.0)	2 (4.0)
Hirose	2020	59	59 (100)	53 ± 18	KDIGO	12 (20.34)	0	1
Kahlberg (RINGER)	2021	13	NR	NR	AKIN	4 (30.77)	1 (7.69)	1 (7.69)
Mascia D	2021	60	14 (23.33)	43.1 ± 3.8	AKIN	13 (21.67)	2 (3.33)	3 (5.0)
Kahlberg (CUSTODIOL)	2021	10	NR	NR	AKIN	5 (50)	0	0
Zarkowsky	2021	81	NR	43 (36–49)	RIFLE	36 (44.44)	12 (14.81)	8 (9.88)
Teter	2022	2812	127	30.1 (±25.8)	CREA increased 0.5 mg/dL	571 (20.30)	119 (4.23)	120 (4.26)
**(b)**								
**Author**	**Year**	**N. Pt**	**Preoperative CKD**	**Renal Ischemia Time**	**Criteria Used for AKI Definition**	**Postoperative AKI** **(N/%)**	**Dialysis** **(N/%)**	**30-Day Mortality** **(N/%)**
Crawford ES	1986	101	18 (17.82)	19 (7–69)	NR	16 (15.84)	8 (7.92)	8 (7.92)
Qvarfordt	1986	77	42 (54.54)	25	Rise in serum creatinin level	18 (23.37)	2 (2.59)	1 (1.29)
Taylor SM	1997	27	3 (11.11)	21 (11–55)	CREA increased 20%	2 (7.40)	1 (3.70)	0
Schneider JR	1997	23	10 (43.48)	22 ± 5	CREA increased 20%	6 (26.08)	0	0
Jean-Claude JM	1999	257	80 (31.13)	31.6 ± 16.7	CREA increased 0.5 mg/d	104 (40.46)	25 (9.72)	15 (5.83)
Giulini SM	2000	56	4 (7.14)	20 (12–35)	CREA increased 0.5 mg/d	8 (14.28)	1 (1.78)	1 (1.78)
Sasaki T	2000	13	0	43.6 ± 22.1	NR	6 (46.15)	1 (7.69)	NR
Shortel CK	2003	112	14 (12.50)	NR	CREA increased >20% or >1.5 in man, 1.3 in woman	14 (12.5)	4 (3.57)	7 (6.25)
West	2006	247	45 (18.22)	23.2 ± 9.7	Postoperative crea > 2 mg/dL	54 (21.86)	9 (3.64)	6 (2.42)
Pearce JD	2007	150	41 (27.33)	30 IQR 18	Postoperative crea > 2 mg/dL	21 (14.0)	10 (6.66)	5 (3.33)
Pichlmaier M	2008	109	38 (34.86)	25.5 (10–86)	CREA increased 0.5 mg/dL	56 (51.37)	13 (11.92)	13 (11.92)
Teter	2022	1344	55 (4.09)	30.1 (±25.8)	CREA increased 0.5 mg/dL	252 (8.96)	63 (2.24)	51 (8.81)

**Table 2:** Comparison between the RIFLE, AKIN, and KDIGO classifications for acute kidney injury.

RIFLE Classification		AKIN Classification		KDIGO Classification		All Three
	Cr/GFR		Cr/GFR		Cr/GFR	UO
R	Cr ≥ 1.5× baseline or GFR reduction > 25% vs. baseline	1	Cr ≥ 1.5× baseline; or increased by ≥0.3 mg/dL	1	Cr ≥ 1.5× baseline within 7 days; or increased by ≥0.3 mg/dL within 48 h	<0.5 mL/kg/h for >6 h
I	Cr ≥ 2× baseline or GFR reduction > 50% vs. baseline	2	Cr ≥ 2× baseline	2	Cr ≥ 2× baseline	<0.5 mL/kg/h for >12 h
F	Cr ≥ 3× baseline; or GFR reduction > 75% vs. baseline; or Cr ≥ 4 mg/dL and +0.5 mg/dL vs. baseline	3	Cr ≥ 3× baseline; or Cr ≥ 4 mg/dL and +0.5 mg/dL vs. baseline; or need for dialysis	3	Cr ≥ 3× baseline; or Cr ≥ 4 mg/dL; or need for dialysis; or In patients < 18 years old, GFR < 35 mL/min/1.73 m^2^	<0.3 mL/kg/h for >24 h; or anuria for 12 h
L	Dialysis for >28 days	/		/		
E	Dialysis for >3 months	/		/		

UO: urine output, Cr: serum creatinine, GFR: glomerular filtration rate. Adapted from Belletti A, Licheri M, Bove T., Perioperative Renal Pharmacological Protection During Cardiovascular Surgery, in Visceral Vessels and Aortic Repair Springer Science and Business Media LLC, 2019.

**Table 4:** Acute kidney injury, dialysis, and 30-day mortality according to perfusion type.

Type of Perfusion	n.	AKI	%	Dialysis	%	30-Day Mortality	%
Ringer	119	33	**27.73**	3	**2.52**	3	**2.52**
Custodiol	81	24	**20.16**	2	**2.46**	3	**3.70**
Other Cold (mannitol/NaCl)	2966	647	**21.8**	133	**4.48**	130	**4.38**
Warm Blood	26	14	**53.84**	0	**0**	1	**3.85**

## Data Availability

The raw data supporting the conclusions of this article will be made available by the authors on request.
